# Supplementary material for: A Culturomics-Based Bacterial Synthetic Community for Improving Resilience towards Arsenic and Heavy Metals in the Nutraceutical Plant Mesembryanthemum crystallinum
Source: Int J Mol Sci. 2023 Apr 10;24(8):7003. doi: 10.3390/ijms24087003 (PMC10138511; doi:10.3390/ijms24087003)
Supplement: Supplementary file 1 [file ijms-24-07003-s001.zip › ijms-2336220-supplementary file S1.pdf]

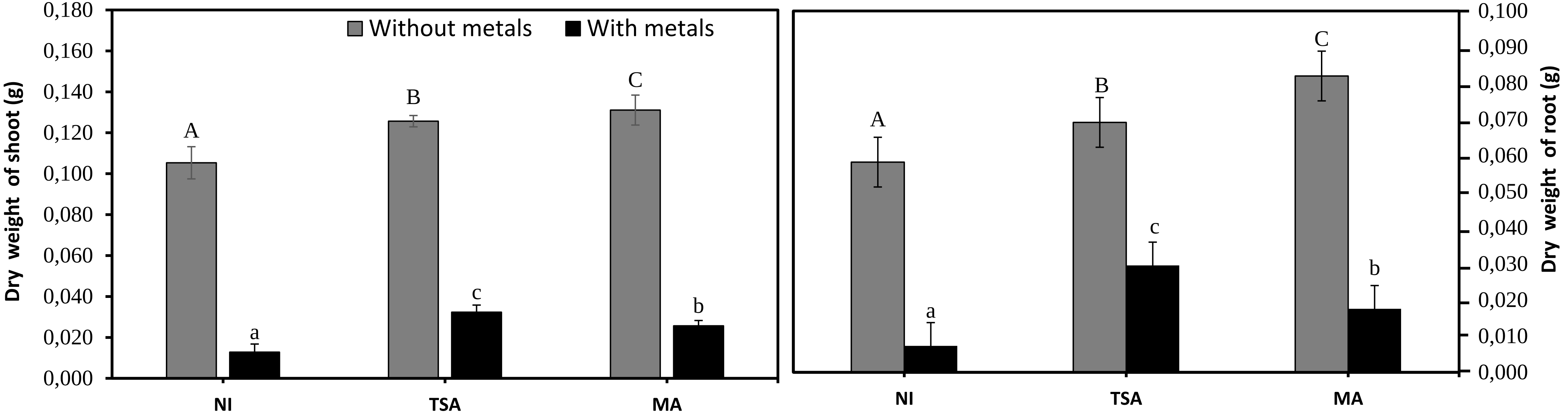

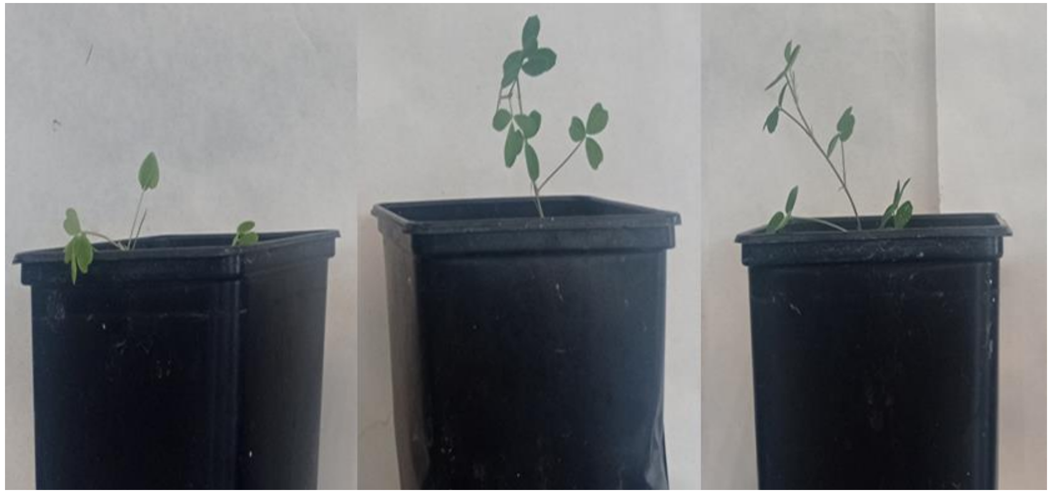

NI TSA MA

Statistically significant differences with regard to the non-inoculated control plants at $p < 0.05$ are indicated by different letters (upper case for the plants grown in the absence of metals and lower case for plants grown in the presence of a mix of metals)

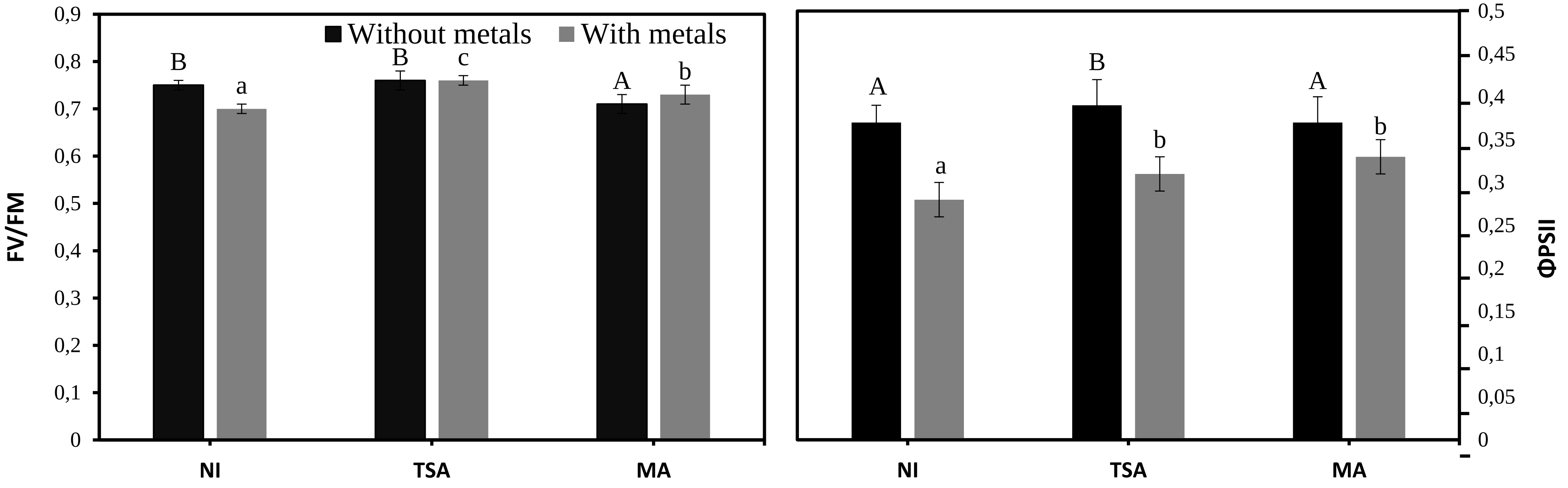

Table S3. Accumulation of metal/loid in shoots and roots of *M. sativa*

Strain	As (mg/Kg)	Cd (mg/Kg)	Cu (mg/Kg)	Zn (mg/Kg)
**Shoot**				
**NI**	15.4545±0.17a	0.3409±0.17a	5.2272±0.23a	28.1818±0.11a
**TSA**	18.2027±0.15b	1.0228±0.02c	5.8755±0.21b	75.2304±0.08b
**MA**	31.1479±0.17c	0.8166±0.01b	10.9659±0.32c	77.6948±0.17c
**Root**				
**NI**	30.9938±0.14a	1.9996±0.00b	23.8952±0.67a	149.4701±0.50b
**TSA**	87.6584±0.22c	3.7638±0.03c	31.2500±1.11c	197.1077±0.72c
**MA**	61.7466±0.22b	1.4325±0.01a	25.0444±0.76b	94.8429±0.34a

Statistically significant differences with regard to the non-inoculated control plants at $p < 0.05$ are indicated by different letters (upper case for the plants grown in the absence of metals and lower case for plants grown in the presence of a mix of metals)
