# Supplementary material for: A Culturomics-Based Bacterial Synthetic Community for Improving Resilience towards Arsenic and Heavy Metals in the Nutraceutical Plant Mesembryanthemum crystallinum
Source: Int J Mol Sci. 2023 Apr 10;24(8):7003. doi: 10.3390/ijms24087003 (PMC10138511; doi:10.3390/ijms24087003)
Supplement: Supplementary file 1 [file ijms-24-07003-s001.zip › ijms-2336220-supplementary table S1 and S2.pdf]

Table S1. Summary of the protocols used for the determination of plant growth promoting properties.

PGP	Medium	Inoculation	Observation	Reference
Phosphate solubilisation	NBRIP agar plates containing $Ca_3(PO_4)_2$ as the sole phosphate source	200 µL of bacterial culture were added to a well excavated in the agar. Plates were incubated at 28 ºc for 5-7 days	The appearance of clear halo around the wells was indicative of P solubilisation. The halo diameter was measured.	(Nautiyal, 1998)
Potassium solubilisation	Aleksandrov medium plates containing insoluble K (mica)	200 µL of bacterial culture were added to a well excavated in the agar. Plates were incubated at 28 ºc for 5-7 days	The appearance of clear halo around the wells was indicative of K solubilisation. The halo diameter was measured.	(Aleksandrov et al., 1967)
Nitrogen Fixation	Medium NFB with no nitrogen source	The bacteria were streaked on the plates and incubated at 28 ºC for 5 -7days	The massive growth of colonies in the plates was indicative of nitrogen's fixation activity.	(Döbereiner, 1980)
Ability to form biofilms	Adhesion to the bottom of 96 wells microtiter plates	Wells containing TSA medium were inoculated with 5 µL of bacterial cultures in quadruplicate. Plates were incubated at 28 ºC for 5 days	After emptying the wells and washing 5 times with water, biofilms were stained with 1% crystal violet for 10 min. After washing again, the dye was solubilized in ethanol:HCl and the absorbance at 595 nm was determined	O'Toole (2011)
Siderophores production	CAS (chromo-azurol-S) agar	200 µL of bacterial culture were added to a well excavated in the agar. Plates were incubated at 28 ºC for 5-7 days	The appearance of a red-orange halo around the well indicated a positive result. The diameter of the halo was measured	(Schwyn & Neilands, 1986)
Auxins (IAA) Production	3 ml of liquid cultures of NB medium (Nutrient Broth, 8 g l^{-1}) containing 1 mM tryptophan	Cultures were incubated at 28 ºC for 48 h at 200 rpm	The cultures were centrifuged, and the amount of IAA produced was determined in 1 ml of the supernatant by addition of 3 ml Salkowski reagent. The absorbance at 535 nm was registered and the concentration of auxins was calculated using a standard curve made with commercial IAA	(Salkowski, 1885)

Table S2. Summary of the protocols used for the determination of enzymatic activities.

Enzymatic activities	Medium	Inoculation	Observation	Reference
Amylase	Starch agar plates	Bacteria were stricken on the plates. The plates were incubated for 5-7 days at 28 ºC	The disappearance of starch around the colonies was revealed by adding lugol (dark blue color)	Navarro-Torre et al., 2020
DNAase	DNAase agar	Bacteria were stricken on the plates. The plates were incubated for 5-7 days at 28 ºC	The activity was revealed by adding 10 ml of HCl 1 N on top of the plate. Appearance of clear zone around the colonies	Navarro-Torre et al., 2020
Protease	Casein agar	Bacteria were stricken on the plates. The plates were incubated for 5-7 days at 28 ºC	The presence of a clear halo around the colony was positive for protease activities.	(Prescott & Harley, 2002)
Lipase	Tween agar	Bacteria were stricken on the plates. The plates were incubated for 5-7 days at 28 ºC	The presence of a precipitate halo around the colonies was positive for lipase activity	(Prescott & Harley, 2002)
Cellulase	Ammonium mineral agar (AMA) containing 1% carboxymethyl cellulose	Bacteria were stricken on the plates. The plates were incubated for 5-7 days at 28 ºC	The activity was revealed by adding 1 mg/mL Congo Red for 15 minutes, followed by 1M NaCl for 15 minutes. Appearance of a clear zone against red background	Elbetagy et al. (2000)
Pectinase	Ammonium mineral agar (AMA) containing 1% pectin	Bacteria were stricken on the plates. The plates were incubated for 5-7 days at 28 ºC	Pectinase activity was revealed by adding 2% CTAB. The appearance of clear halo around the colonies indicated pectinase activity	Elbetagy et al. (2000)
Chitinase	Mineral medium supplemented with 1.5% colloidal chitin	Bacteria were stricken on the plates. The plates were incubated for 5-7 days at 28 ºC	The appearance of clear halos around the colonies was indicative of chitinase activity	Mesa et al. (2015a)

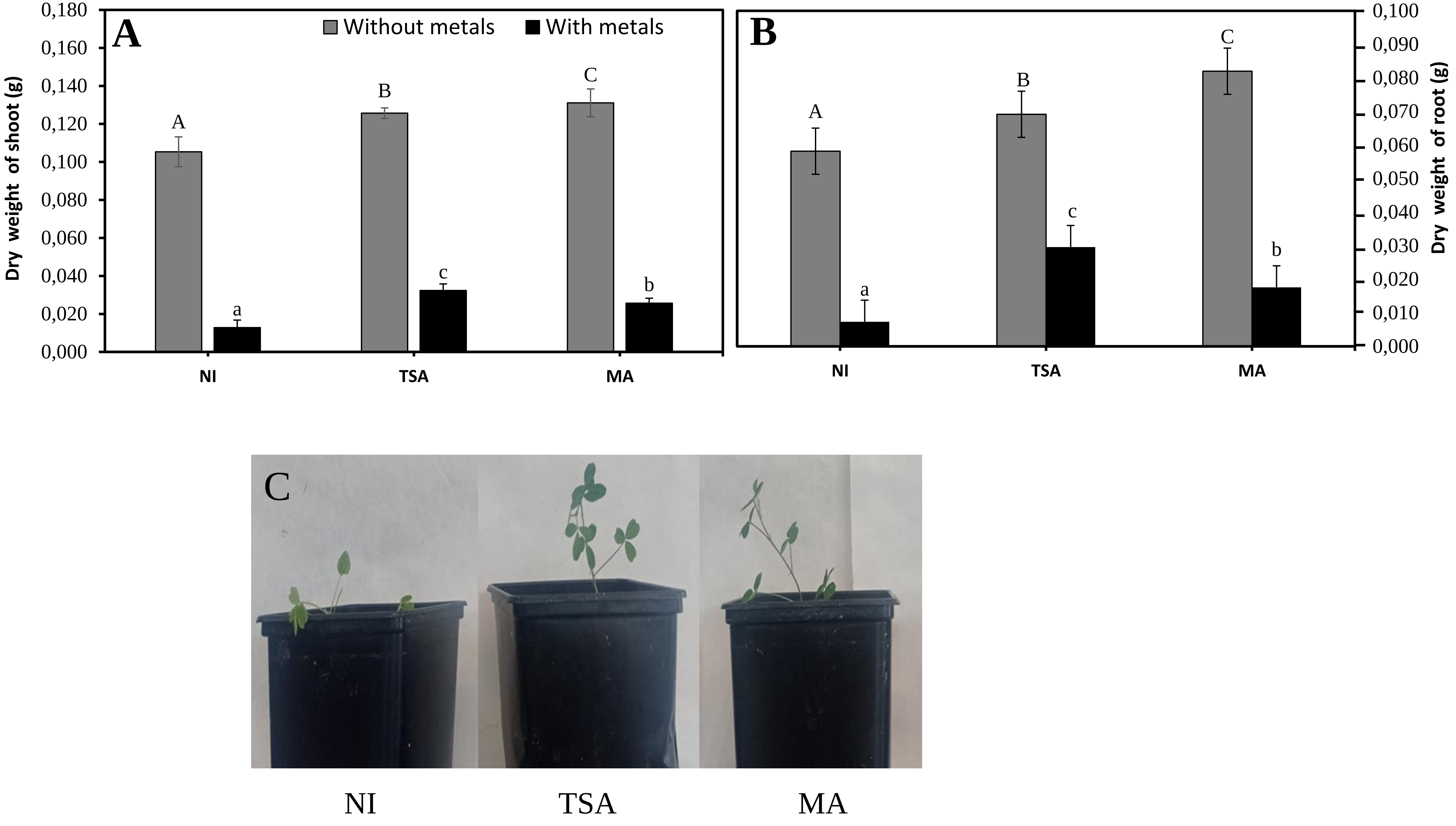
A
Without metals
With metals
Dry weight of shoot (g)
0,180
0,160
0,140
0,120
0,100
0,080
0,060
0,040
0,020
0,000
A
a
B
c
C
b
NI
TSA
MA
B
Dry weight of root (g)
0,100
0,090
0,080
0,070
0,060
0,050
0,040
0,030
0,020
0,010
0,000
A
a
B
c
C
b
NI
TSA
MA
C
NI
TSA
MA

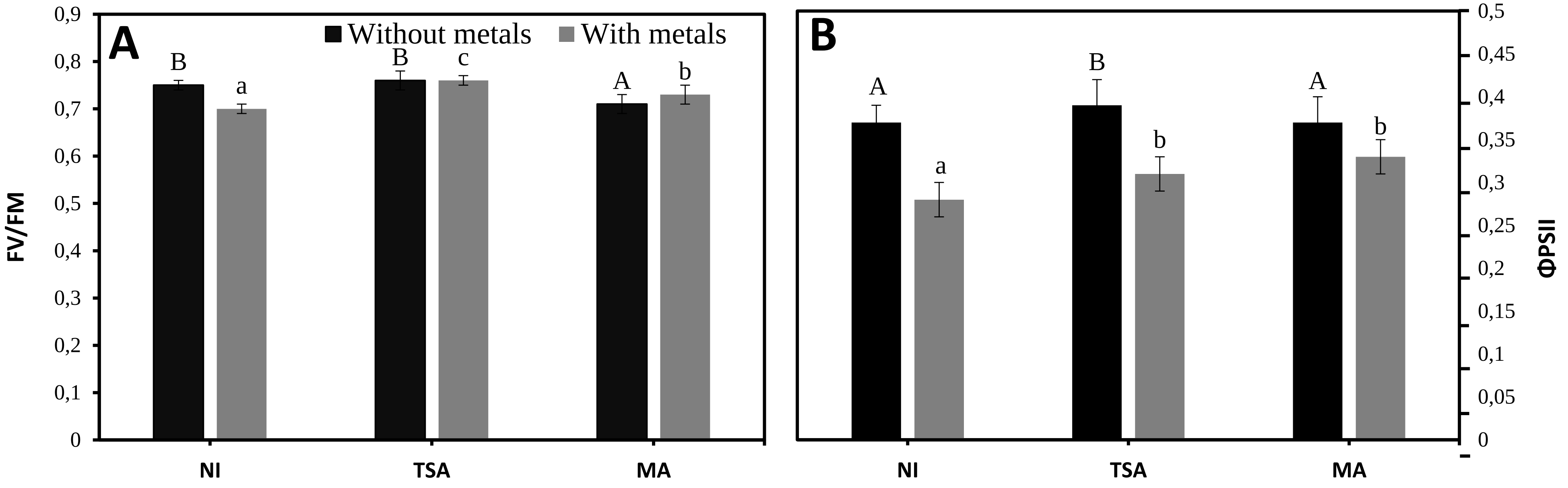

Table S3. Accumulation of metal/loid in shoots and roots of *M. sativa*

Strain	As (mg/Kg)	Cd (mg/Kg)	Cu (mg/Kg)	Zn (mg/Kg)
**Shoot**				
**NI**	15.4545±0.17a	0.3409±0.17a	5.2272±0.23a	28.1818±0.11a
**TSA**	18.2027±0.15b	1.0228±0.02c	5.8755±0.21b	75.2304±0.08b
**MA**	31.1479±0.17c	0.8166±0.01b	10.9659±0.32c	77.6948±0.17c
**Root**				
**NI**	30.9938±0.14a	1.9996±0.00b	23.8952±0.67a	149.4701±0.50b
**TSA**	87.6584±0.22c	3.7638±0.03c	31.2500±1.11c	197.1077±0.72c
**MA**	61.7466±0.22b	1.4325±0.01a	25.0444±0.76b	94.8429±0.34a
